# Provider Perspectives on Analgesic Use in Intrauterine Device Insertion Procedures: A Mixed Methods Analysis

**DOI:** 10.7759/cureus.56580

**Published:** 2024-03-20

**Authors:** Camryn Daidone, Kaitlyn Morris, James Colquitt, Gwenn Jackson

**Affiliations:** 1 Research, Edward Via College of Osteopathic Medicine, Monroe, USA; 2 Obstetrics and Gynecology, Edward Via College of Osteopathic Medicine (VCOM) Louisiana, Monroe, USA

**Keywords:** general gynecology, public health education, contraceptive methods, multi-modality pain management, long-acting reversible contraception (larc)

## Abstract

Context: The intrauterine device (IUD) is one type of long-acting reversible contraceptive that is becoming increasingly popular among patients and healthcare providers alike, though many are deterred from using this option due to pain or fear of pain with IUD insertion. While the IUD insertion process itself is standardized, the use of pain medication is not. There is a lack of research regarding provider preference in analgesic use for IUD insertion procedures, which analgesics are being provided to patients, and under which circumstances. This study aims to explore which analgesics are being used routinely in clinical settings, which patient populations are more likely to receive or benefit from these treatments, and why. Secondarily, this study aims to evaluate the impacts of provider characteristics such as location of training and practice, length of practice, and type of training in analgesic administration.

Methods: Various national organizations were contacted via email and asked to distribute the “IUD Pain Management” survey via discussion board or email newsletter. This survey was developed to gather demographic information on providers of IUD placement procedures and evaluate trends in analgesic methods used based on provider and patient characteristics. Additionally, the survey included an opportunity for participants to agree to participate in a brief interview to further elaborate on their responses via phone. Survey responses were collected and evaluated on the secure QuestionPro platform. Results from the interview were qualitatively assessed by coding recurrent themes between participant interviews.

Results: Survey respondents represented physicians from family medicine and OB-GYN specialties, as well as nurse practitioners, registered nurses, physician assistants, and OB-GYN resident physicians. The average length of clinical practice is 6.7 years. The majority of respondents reported offering some sort of analgesic for IUD insertion procedures, with nonsteroidal anti-inflammatory drugs being the most commonly used. Participants also reported an increased likelihood of prescribing analgesics for adolescent and nulliparous patients. Participant interviews included themes such as patient perception of pain, provider training, barriers to access, and alternative analgesic options.

Conclusions: Our study has identified a significant amount of variation in practices regarding analgesic use for IUD insertion procedures and highlighted some underlying causes of these inconsistencies. Future studies should further investigate trends in analgesic administration in IUD insertion procedures with a larger sample size and delve into factors such as provider education and barriers to access.

## Introduction

The intrauterine device (IUD) is a type of long-acting reversible contraceptive that is becoming increasingly popular among patients and healthcare providers alike, as they are effective in preventing pregnancy, cost-effective, and their effectiveness is not dependent on patient compliance. Levonorgestrel IUDs provide a progesterone-only option with demonstrated effectiveness in treating heavy menstrual bleeding, among other hormonal symptoms [1], and the copper IUD provides a long-acting reversible contraceptive option for patients who prefer not to take hormones or for which hormonal contraceptives may be contraindicated [1,2]. While the IUD has many benefits for patients, many are deterred from using this option due to pain or fear of pain with IUD insertion [2], which includes a speculum exam, uterine sounding, tenaculum placement, and insertion of the device through the cervix and into the body of the uterus. While the procedure itself is standardized, the use of pain medication is not.

Pain reduction therapies for IUD insertion have been widely investigated with mixed results. Studies have reported lidocaine paracervical block [3,4], vaginal or orally administered misoprostol or other prostaglandins [5], and non-steroidal anti-inflammatory drugs [6,7] as effective. Other studies investigating the use of the same analgesic drugs found no efficacy [8-15]. It is also important to note that the use of analgesics may be dependent on the demographic of the patient, with potentially increased efficacy in younger patients [7], those with a recent history of other gynecologic procedures such as intrauterine aspiration [12], and in nulliparous patients who may experience increased pain [12].

Additionally, psychosocial elements play a role, as patient anxiety could be an important predictor of pain perception during the procedure [14]. The literature is clearly divided on the efficacy of analgesics in pain reduction during IUD insertion procedures, and the debate on the different types of analgesics as well as patient characteristics is complex, leaving no true standard of care. Thus, the decision to administer analgesics during IUD insertion procedures is left to providers. However, there is a lack of research regarding provider preference in analgesic use for IUD procedures, which analgesics are being provided to patients, and under what circumstances.

Through collection of survey data and interviews with IUD insertion providers, this study intends to address the lack of standardization of care by investigating which analgesics are being used routinely in clinical settings, which patient populations are more likely to receive or benefit from these treatments, and why. Secondarily, this study aims to evaluate whether trends in analgesic administration are impacted by provider characteristics such as location of training and practice, length of practice, and type of training.

## Materials and methods

The “IUD Pain Management Survey” was created and conducted by researchers at the Edward Via College of Osteopathic Medicine, Louisiana Campus. Our survey was designed to answer the following questions: What analgesics are currently being used in clinical settings? (1) Which patient populations are more likely to receive treatment or benefit from treatment? (2) How do provider characteristics impact trends in analgesic administration during IUD insertion? (3) (Appendix 1). This survey included several demographic questions related to training, including type (physician, resident physician, nurse practitioner, etc.) and location, as well as location and length of current practice. Questions designed to gauge current use of specific analgesics (NSAIDS, lidocaine gel, paracervical block, misoprostol, and non-pharmacologic therapies) and specific patient characteristics that might influence the decision to administer analgesics, such as nulliparity, adolescent age, and history of recent gynecologic procedures, were included in the survey.

The survey was sent to various national organizations, including the American College of Osteopathic Obstetricians and Gynecologists (ACOOG), the American College of Obstetricians and Gynecologists (ACOG), Bayer Women’s Health Care, and the National Nurse Practitioner Association (NNPA), via email with a request to distribute the survey via email and/or discussion board post to organization members. Study data were collected and managed using the secure QuestionPro survey managed by faculty researchers at the Edward Via College of Osteopathic Medicine. All survey data was de-identified, and descriptive statistics were evaluated for general trends.

Survey participants were given the opportunity to participate in a follow-up interview and provide contact information. The semi-structured interview aimed to identify areas to improve access to analgesics, the provider’s satisfaction with current analgesic options, and the provider’s personal standard of care. Interviews lasted between 10 and 40 minutes, depending on how much the participant wished to elaborate on their responses. The complete interview script can be viewed in Appendix 2. The interviews were recorded and transcribed through an online transcription service before the qualitative analysis of de-identified participant responses. The recordings of the interviews were deleted when transcription was complete. Transcripts from the semi-structured interview were coded manually by researchers at the Edward Via College of Osteopathic Medicine to identify recurrent words and phrases. Quotes from participant interviews were placed within identified common themes extrapolated from the interviews. Common themes were occasionally broken down into sub-themes if certain concepts were recurring within a particular common theme.

The Institutional Review Board at the Edward Via College of Osteopathic Medicine approved the survey and semi-structured interview.

## Results

Survey

Our survey had a total of 52 responses; of these respondents, 33 were currently providing IUD insertion procedures as part of their clinical practice and were eligible to complete our survey.

Participant demographics

Clinical Practice

**Figure 1:**
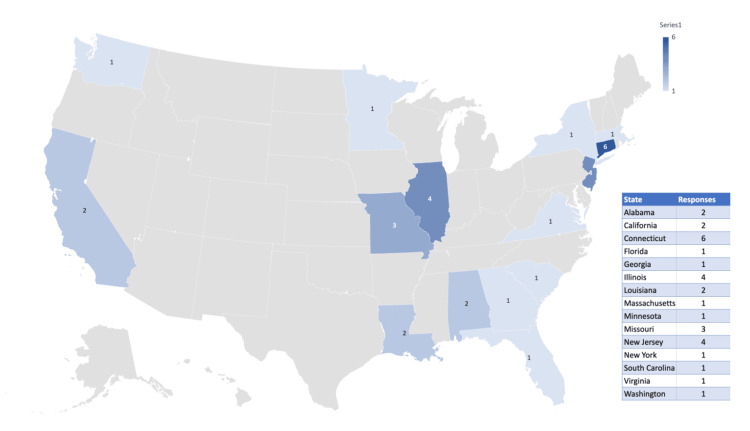
A map indicating which states in the United States of America are currently represented in this sample’s clinical practice location. Note: This excludes all international responses.

The length of clinical practice for survey respondents ranged from <1 year to 32 years, with an average length of 8.12 years. These respondents are currently practicing in 15 states, as indicated in Figure 1. Additionally, we received international responses from participants practicing in Spain, Nigeria, and Malawi. As all international respondents indicated that they received their medical training in the United States, we included their responses in our study.

Training

**Figure 2:**
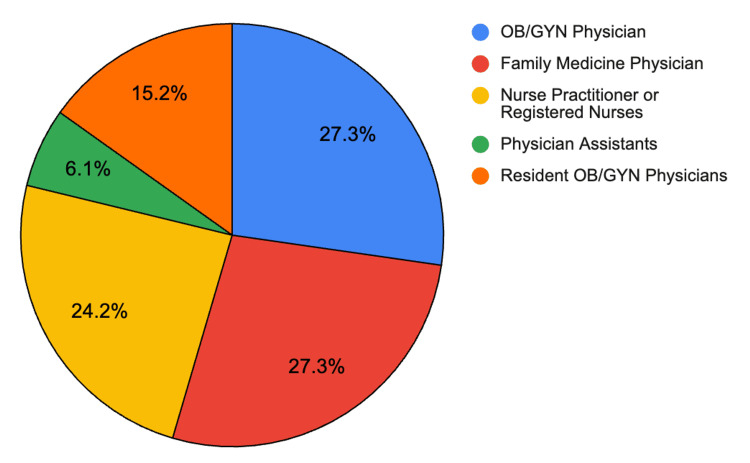
A pie chart indicating the type of training reported by survey respondents

The majority of respondents are practicing OB-GYN physicians (27.27%) (D.O. or M.D.) or practicing family medicine physicians (27.27%) (D.O. or M.D.), 24.24% of respondents are nurse practitioners (NP) or registered nurses (RN), 6.06% are physician assistants, and 15.15% of respondents are physicians currently receiving residency or fellowship training. Of the physicians completing training, all were OB-GYN residents; 50% are completing post-graduate year two, and 50% are completing post-graduate year three. The reported type of training among participants is indicated in Figure 2.

**Figure 3:**
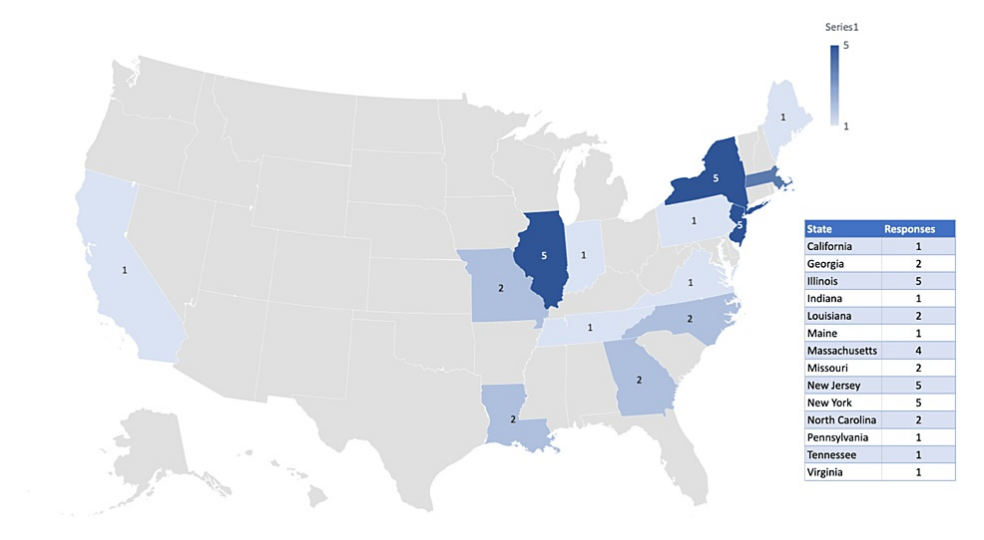
A United States map indicating the location of training for survey respondents

Respondents reported receiving their medical training in 13 states, as indicated in Figure 3. No respondents indicated that they received their training outside of the United States.

Analgesic Use

When asked about provider-perceived patient anxiety, 67.74% of respondents reported that “a majority of my patients are worried about pain with this procedure.” One respondent reported, “my patients are not worried about pain with IUD placement.”

**Figure 4:**
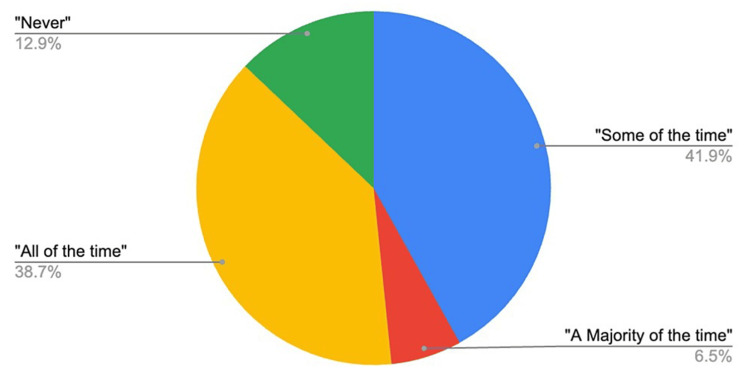
A pie chart indicating the overall frequency of reported use of analgesics in IUD insertion procedures

When asked if they provide analgesic options for their IUD insertion procedures, 41.94% responded that they offer analgesic options “some of the time,” and 38.71% stated that they offer analgesics “all of the time.” 2 respondents reported that they offer analgesics “a majority of the time,” and 12.90% reported that they never offer analgesic options. The frequency of reported analgesic use is shown in Figure 4.

The following analgesics were represented in this sample: NSAIDS (39.68%), paracervical block (20.63%), misoprostol (12.7%), lidocaine gel (6.35%), and others (14.29%). For participants who selected “other” analgesics, the following options were reported: diazepam, nitrous oxide, IV sedation, alprazolam, and topical benzocaine for topical lidocaine spray prior to paracervical injections.

With regard to over-the-counter pain medications such as NSAIDs, 80% of respondents reported that they “always recommend that patients take over-the counter pain relievers like NSAIDS for post-procedure pain,” and 20% reported that they recommend patients take over-the-counter pain relievers “a majority of the time.”

With regard to the frequency of analgesic use, 17 respondents reported that they “always” offer NSAIDS to patients, five respondents reported that they “sometimes” offer misoprostol, and seven respondents indicated that they “sometimes” offer paracervical block. Ten participants reported that they “always” recommend non-pharmacologic pain management options, while eight noted that they “sometimes” offer non-pharmacologic pain management options.

When asked to rank, on a scale of 0-10, their likelihood of providing analgesics to specific patient populations, respondents reported that they are most likely to provide analgesic options for adolescents (average: 8.71/10) and nulliparous patients (average: 8.23/10). They also reported that they would be likely to provide analgesics to patients with a recent history of gynecologic procedures such as dilation and curettage (average: 7.30/10). Participants also reported some likelihood of providing analgesic options for multiparous patients (average: 6.68/10).

Structured interview results

Six providers participated in the interview portion of the study. The interview length ranged between 10 and 40 minutes. 

Themes/subthemes identified

Major recurring themes identified between patient interviews were patient population perception of pain, misoprostol, provider training, paracervical blocks, barriers to access, use of different analgesics/tools, satisfaction with current analgesics, and nonpharmacologic pain relief options. Within the common themes, further subthemes were identified that pertained to the recurrent common themes. Subthemes are placed in bullet points below common themes in Table 1. For the complete qualitative analysis, including participant quotes, refer to Appendix 3.

**Table 1:** Themes and subthemes from semi-structured interviews

Themes	Subthemes
Patient population perception of pain	Barriers to access
Use of misoprostol	Potential loss to follow up
Provider training	Stigma
Pain relief education with abortion training	Lack of adequate analgesic options
Paracervical blocks	Use of other analgesics (NSAIDS, nitrous oxide, sedation, benzodiazepines)
Nonpharmacologic options (patient education/talk anesthesia, hot packs)	Use of tools (atraumatic tennaculum, ultrasound guidance)

## Discussion

This is a mixed-methods study that includes a survey component as well as a semi-structured interview to answer three primary research questions: (1) What analgesics are currently being used in clinical settings? (2) Which patient populations are more likely to receive treatment or benefit from treatment? (3) How do provider characteristics impact trends in analgesic administration during IUD insertion?

Survey

Our data sample of 36 survey responses represents a variety of providers by training and location. While the majority of our sample includes physicians, there is equal representation from both family medicine and obstetrics/gynecology specialties, as well as significant representation from nurse practitioners, registered nurses, and physician assistants. These participants currently practice in 15 states across the United States of America and three other countries. The states represented are predominantly situated on the east coast and in the midwest. Due to variation in socioeconomic climate and legislation by state, as well as a lack of standardization in training and procedures for analgesic use in IUD insertion procedures represented in the literature, we believe that there may be some variation in practice by region. Future studies should seek to evaluate regional trends in analgesic use.

Respondents reported a length of practice anywhere from <1 year to 32 years, with the average length of practice being 8.12 years. The average length of practice for this sample is less than 10 years, indicating that our sample is biased towards providers who are newer in practice and includes five OB-GYN resident physicians. Future studies should evaluate whether the length of practice impacts analgesic use.

The majority of our respondents reported offering some type of analgesic for IUD insertion, and NSAIDs were the predominant form of analgesic offered (39.68%). This is consistent with current research that supports the efficacy of NSAID use in IUD insertion [4,5]. Additional analgesics utilized were paracervical block, misoprostol, and lidocaine gel. Interestingly, 14.29% of respondents reported using sedatives that were not represented in our survey, including benzodiazepines such as diazepam (Valium, Genentech) and alprazolam (Xanax, Pfizer), nitrous oxide, and IV sedation. Also mentioned was topical benzocaine for topical lidocaine spray before paracervical injections. This data highlights the lack of a gold standard in analgesic use for IUD insertion procedures and indicates that providers are using their best clinical judgment as opposed to following evidence-based care plans.

These alternative options, such as nitrous oxide, benzodiazepines, and IV sedation, are not well studied in the current literature. The use of benzodiazepines in IUD insertion procedures may be beneficial, as 67.74% of survey respondents report that “a majority” of their patients are worried about the pain associated with the IUD insertion procedure. Though benzodiazepines may not treat the somatic pain associated with the procedure, their ability to help manage patient anxiety may provide substantial benefit to patients. The additional structured interview portion of this project aims to further evaluate the themes surrounding the use of analgesics that are less common or best suited for specific patient populations.

When prescribing analgesics for specific populations, respondents reported that they would be more likely to use analgesics for adolescent patients (8.75 out of 10) than nulliparous (8.23 out of 10). Respondents are less likely to offer analgesics to multiparous patients (6.68 out of 10) and may use analgesics in patients with a recent history of gynecologic procedures (7.3 out of 10). These responses are consistent with current literature that suggests the efficacy of analgesic use in adolescent patients [7], as well as nulliparous patients and those who have undergone recent gynecologic procedures [12].

The survey component of our study is limited primarily by a small sample size. The mean length of practice for the providers in our study was <10 years, indicating that most respondents were not long out of clinical training. Still, our survey population includes providers with a variety of training spanning 15 states across the United States plus three other countries and provides valuable insight into trends in analgesic use in IUD insertion procedures. Additionally, our study offered an opportunity for survey respondents to elaborate on their responses in the form of a semi-structured interview.

Semi-structured interview 

The semi-structured interview elicited a wide variety of responses in terms of what providers currently utilize for in-office pain management, further affirming the idea that there is a lack of standardization in providing pain relief for IUD placement procedures.

The first common theme to emerge was patient populations' perceptions of pain. The overall consensus was that the three parts of IUD insertion that are most painful for patients are tenaculum placement, sounding the uterus, and the insertion itself. Providers often state that there is a wide variety of patient pain responses to IUD insertion, particularly with tenaculum placement. Some patients tolerate tenaculum placement very well, and for other patients, tenaculum placement is the most painful part of the procedure. The variety of responses between patients further emphasizes the need to standardize the use of analgesics with IUD insertion.

Providers also stated that patients' past experiences with gynecologic care can inform how they respond to pain. If a patient had a traumatic experience surrounding gynecologic care in the past, providers stated that anxiety toward the IUD insertion procedure itself is a problem that needs to be addressed. Other providers stated that their patients can tolerate the procedure because it is a pain that they have experienced before, particularly if they have problems with cramping and painful menstruation. Providers also echoed similar sentiments, saying that many of their patients would be great candidates for IUDs but are not interested in them because they fear how painful IUD insertion is. Standardization in analgesic utilization could increase access and usage of IUDs among patients who opt for alternative contraception out of fear of pain with IUD insertion.

The next theme to emerge from the interviews was provider training. Providers indicated that they had little to no formal training on providing different types of pain relief for IUD insertion during their residency.

Either they learned through a complex family-planning fellowship, where post-abortion IUD insertion was offered, or through clinical experience. A sub-theme that emerged within provider training was pain management through abortion care. It may be valuable to study the curriculum surrounding pain management in abortion training and complex family planning fellowships. Perhaps implementing a similar curriculum in IUD insertion training will increase the provider's repertoire in pain management for these procedures.

The major recurrent theme through all provider interviews was the use of paracervical blocks. There was a wide variety of providers’ attitudes towards the efficacy of paracervical block, their patient populations’ attitudes toward paracervical block, and whether the benefits of the paracervical block outweighed the challenges. The usage and offering of a paracervical block were also widely varied between providers. While a majority of providers interviewed at least offered paracervical blocks to their patients, one provider stated that she does not utilize paracervical blocks for pain management. Another provider stated that she uses paracervical blocks for every single IUD insertion she performs. Overall, there was a wide range of differences between providers utilizing paracervical blocks for IUD insertion.

Studies have shown that a buffered 20 cc 1% lidocaine paracervical block is indeed a beneficial pain relief option, but providers have varying attitudes toward efficacy [3]. One provider stated that she feels like the paracervical block is more painful for her patients and adds unnecessary time to the procedure. Others stated that, anecdotally, they believe their patients who have a paracervical block seem to tolerate the procedure better than patients who opt out of getting a paracervical block. Providers also stated that although they offered a paracervical block, they have difficulty getting buy-in from their patients because of fear of needles and fear of pain from the paracervical block itself. Studies have shown that the paracervical block is indeed a beneficial pain relief option, but providers have varying attitudes toward its efficacy. Perhaps the performance of paracervical blocks and buy-in from patients can be increased by utilizing the paracervical block with other methods of analgesic. One provider stated that before she performs her blocks, she uses a spray of lidocaine gel on the cervix, typically used for dental procedures. She said that this helps ease patients' fear of needles with the paracervical block and that, anecdotally, it seems to work well for her patient population.

Others expressed frustration at the lack of training on how to perform an effective and efficient paracervical block. One provider believes that a lack of adequate training causes clinicians to perform insufficient paracervical blocks. She believes that if someone is trained to do an IUD insertion, they should also be trained in performing paracervical blocks and that providers should not be trained in one or the other, but rather training for IUD insertions and paracervical blocks should go hand-in-hand.

Providers talked about barriers to providing certain analgesics, such as potential loss of follow-up, lack of training, and stigma surrounding women’s pain. Logistically, it can be difficult to give certain pain relief options, such as light sedation or benzodiazepines for anxiety. Providers stated that patients who come in seeking an IUD want it that day, and delaying insertion to provide certain pain relief options could result in a potential loss of follow-up. For example, the use of benzodiazepines before IUD insertion is a pain and anxiety relief option that some providers utilize. However, the use of benzodiazepines requires patients to secure a benzodiazepine prescription and coordinate transportation to and from the clinic. This results in logistical issues, as patients will need to come back another day for the IUD insertion and creates a situation that could result in a potential loss of follow-up. Other providers stated that offering other analgesic methods like the use of light sedation is not logistically possible because if the clinic does not have the capability/resources, or the provider must refer the patient to a different clinic that has the ability to utilize light/moderate sedation, once again creating a situation that leaves patients vulnerable to a potential loss of follow-up.

Stigma surrounding women’s pain was another barrier to patients accessing analgesics for IUD insertions. Providers expressed frustration that women’s pain is just accepted as part of the IUD insertion, and because of this, few patients are offered adequate pain relief options. Others have said that female anatomy is thought to be outside the scope of routine primary care, requiring more specialized OB-GYN care. However, IUD insertions are not just performed at gynecology offices. In fact, IUDs are often placed in the primary care setting. Providers believe that because of this bias, there is little training in analgesic use, such as a paracervical block, in the primary care setting or when training in primary care.

Providers also stated that they wished for more options for pain relief to be available in their clinic, primarily the use of nitrous oxide. Although current data is mixed regarding the efficacy of the use of nitrous oxide for pain relief during gynecological procedures, providers believe that nitrous is another tool they can use to make patients more comfortable during IUD insertions [16]. Furthermore, the use of nitrous for pain relief addresses the logistical problems seen with light sedation. The use of nitrous is a good alternative to light sedation because there isn’t a need for an in-office anesthesiologist.

Overall, the most prominent form of pain management used by providers was NSAIDs. However, many providers say that the use of NSAIDs does not help their patients with pain during the procedure; rather, onboarding their patients with NSAIDs before the IUD insertion was helpful with post-procedural pain. Many stated that a major barrier was the ability to have their patients come to the clinic with an NSAID onboarded already, either because they were not able to tell their patient to take NSAIDs before their IUD insertion or because the first time the provider saw them in the clinic was when the patient arrived for their IUD insertions. Many patients have access to their electronic health records through programs such as MyChart. Perhaps one method to address onboarding with NSAIDs before IUD insertion would be to contact patients through their electronic health record or email and instruct them on taking NSAIDs before coming to the clinic.

Finally, providers emphasized the importance of non-pharmacologic options, particularly the use of trauma-informed gynecologic care and “verbal anesthesia." Providers stated that walking patients step-by-step through the procedure and allowing the patient to direct and take charge of their own care makes a huge difference with IUD insertion anxiety. Trust between providers and patients, facilitated when patients are able to openly and honestly communicate with their provider, is critical to addressing procedural anxiety and helping redirect pain during the procedure.

Our study is unique in its ability to allow participants to further elaborate on their responses in the form of open-ended survey questions and a phone interview. While other studies have evaluated objective outcomes of pain management per patient pain scale, this study is one of the first to look at the provider’s opinions and gauge which analgesics and techniques are actually being used in practice. Limitations of this study include a small sample size for both the survey and interview responses and a limited range of geographic areas and types of training represented in our sample. Future studies should seek to evaluate trends in analgesic use in IUD insertion procedures among a larger population of providers. Additionally, our study included five responses from OB-GYN residents and calls for more research on the education currently being provided to residents regarding analgesic use in IUD insertion procedures.

This study further emphasized the need for standardization of analgesic use surrounding not just IUD insertions but in-office gynecological care as a whole. The providers interviewed helped give vital insight into the current practice for pain relief with IUD insertions. Providers also gave numerous suggestions for areas to address to increase the use of analgesics, such as training in paracervical blocks in tandem with IUD insertion training, using nitrous oxide in-office, and utilizing trauma-informed and patient-directed care during procedures.

## Conclusions

This mixed-methods study highlights the current landscape of analgesic use during intrauterine device (IUD) insertion procedures and addresses the lack of standardization in clinical care. The survey component, with 52 responses and 36 eligible participants, reveals a diverse provider population practicing across 13 states. Notably, the majority of respondents offer some form of analgesia, with NSAIDs being the predominant choice. However, the study uncovers variations in practice, highlighting the absence of a gold standard in analgesic use. The semi-structured interviews further emphasize the complexity of provider decision-making, revealing themes such as patient population perceptions of pain, provider training, and barriers to access. Paracervical blocks emerge as a significant topic, with varying attitudes surrounding their efficacy and usage. This study advocates for standardization and enhanced training, emphasizing the importance of addressing patient anxiety and incorporating trauma-informed care. While the study's limitations include a small sample size and limited geographic representation, it provides valuable insights into the nuances of analgesic practices during IUD insertions, paving the way for future research to explore regional trends and educational gaps among providers. Ultimately, this research contributes to the ongoing dialogue on optimizing pain management strategies in gynecological procedures and calls for a comprehensive approach to enhance patient experiences and outcomes.

## Figures and Tables

**Figure 5:**
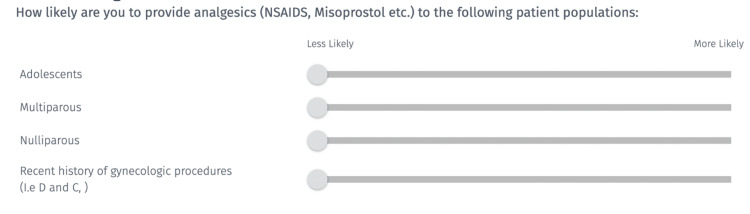
Survey Question 8

## Appendices

Appendix 1 

Survey Questions

1.     Do you currently provide IUD insertion procedures as a part of your clinical practice

·       Yes

·       No

If no, end of survey. If yes, proceed to question 2.

You have been asked to complete this survey as part of a research project conducted by Dr. Gwenn Jackson, MD, Dr. James Colquitt, PhD, and Student Doctors Kaitlyn Morris and Camryn Daidone at Edward Via College of Osteopathic Medicine-Louisiana. The research project is called “Provider perspectives on analgesic use in intrauterine device insertion procedures” and is designed to determine what analgesics are being used routinely in clinical settings with IUD insertion procedures and provider trends in analgesic administration. Your responses are entirely voluntary, and you may refuse to complete any part or all of this survey. This survey is designed to be confidential. Meaning that if you voluntarily offer personal or contact information in the final question regarding a follow-up interview, we will be collecting personally identifiable information. This information will be removed from the survey data and kept confidential. It will not be disclosed by the research team, except when required by law or by the VCOM Institutional Review Board. By completing and submitting the survey, you affirm that you are at least 18 years old and that you give your consent for Kaitlyn Morris and Camryn Daidone to use your answers in their research. If you have any questions about this research before or after you complete the survey, please contact Kaitlyn Morris at kmorris@ulm.vcom.edu, Camryn Daidone at cdaidone@ulm.vcom.edu or Dr. Gwenn Jackson at gjackson@ulm.vcom.edu. If you have any concerns or questions about your rights as a participant in this research, please contact the Chairman of the VCOM IRB, Dr. Gunnar Brolinson, at (540) 231-4981 or pbrolins@vcom.vt.edu.

2.     Have you read the above consent statement and agree to participate in the survey?

·       Yes

·       No

If no, end of survey. If yes, proceed to question 3.

3.     How long have you been in your medical practice? (text box)

4.     Which of the following states do you practice in? (drop down menu)?

5.     In which state were you trained/completed residency (drop down menu)?

6.     Which of the following best describes your current training

·       Resident/Fellowship physician

·       OB/GYN (M.D. or D.O.)

·       Family medicine physician (M.D. or D.O.)

·       RN or Nurse practitioner

·       Physician Assistant

·       Other

7.     How often do you offer analgesics for your patients with IUD insertion procedures?

·       Never

·       Some of the time

·       A majority of the time

·       I always offer analgesics for my patients undergoing IUD insertion procedures

8.     Sliding scale:

9.     For patients who decide to use an IUD for contraception, which of the following best describes your patients' attitudes toward pain with IUD placement prior to the procedure?

·       My patients are not worried about pain with IUD placement

·       Some of my patients are worried about pain with the procedure

·       A majority of my patients are worried about pain with the procedure

·       Pain with the procedure is a large worry for nearly all of my patients

10.  Which of the following do you prescribe in your current practice for IUD insertion? (Select all that apply)

·       Lidocaine Gel (if checked, proceed to question 14)

·       Misoprostol (if checked, proceed to question 11)

·       NSAIDs (if checked, proceed to question 12)

·       Opioids

·       Paracervical block (if checked, proceed to question 13)

·       Tramadol

·       None of the above (if checked, proceed to question 15)

·       Other

11.  How likely are you as a provider to prescribe a cervical softener like misoprostol for your patients prior to IUD placement?

·       Never

·       Sometimes

·       A majority of the time

·       I always prescribe a cervical softener for patients prior to IUD placement

12.  How likely are you as a provider to recommend that patients take over-the-counter pain relievers like NSAIDs for IUD placement pain and post-procedure pain?

·       Never

·       Sometimes

·       A majority of the time

·       I always recommend that patients take over-the-counter pain relievers like NSAIDs for IUD placement pain and post-procedure pain

13.  How likely are you to use a paracervical block for pain during IUD placement procedures?

·       Never

·       Sometimes

·       A majority of the time

·       I always use a paracervical block for tenaculum placement pain during IUD placement

14.  How likely are you to use lidocaine gel for pain during IUD placement?

·       Never

·       Sometimes

·       A majority of the time

·       I always use lidocaine gel for pain during IUD placement

15.  How likely are you to recommend non-pharmacologic pain management options to your patients for pain during IUD placement, such as relaxation techniques, distraction methods, or aromatherapy?

·       Never

·       Sometimes

·       A majority of the time

·       I always recommend non-pharmacologic pain management options to my patients for pain during IUD placement

15.  Would you be willing to be interviewed via phone for this research?  We would email you to schedule a recorded call. During the recorded call you would provide verbal consent to participate in the interview.

·       Yes

·       No

If no, end of survey. If yes, proceed to question 16

16.  Thank you for your interest in participating in a telephone interview. This interview will last ~10 minutes. You will be audio recorded. The recordings will not contain any personal content. Recordings will be used for our data sets. The recordings will be deleted within one week later. If you do not want to be taped, you will not be able to participate. Please indicate below whether you would be willing to participate in an audio recorded phone interview and provide your best contact information (email address and/or phone number).

·       I consent to this interview being audio recorded for transcription purposes

·       I do NOT consent to this interview being audio recorded for transcription purposes

Please provide best contact information (email address and/or phone number) Text box

17.  By submitting this survey, I am consenting for my answers to be used in the study mentioned above. I am submitting this survey voluntarily. I understand that there is a small risk for my information to be tracked back to me. However, I understand that my information will be de-identified and results will be kept in a secure folder.

·       I agree

·       I do NOT agree

Appendix 2

*Interview Script* 

Keep demographic information with structured interviews to look at themes based on region, training, years of practice, etc. 

In your experience, how often do patients ask you about treatment options for pain related to IUD placements? 

In your patients who opt for IUD placements, do you find that they are worried about pain prior to IUD placement?

Do you ever find that patients will opt for other contraception methods besides IUDs because they are worried about how painful the procedure will be? 

Nitrous oxide 

Barriers to care 

Time constraints with providing IUDs? 

Do your patients often report pain during the IUD placement procedure and if so, when in the procedure do you feel that patients are more likely to experience pain

If they have indicated misoprostol use in survey → Do you find that misoprostol increases ease of procedure for IUD placement? 

Did you receive training on providing pain relief during IUD placement, and if so, what types of pain relief options were recommended?

Are you currently satisfied with available pain relief options for IUD procedures 

What would you like to see in field of pain relief that would allow you to be satisfied with pain relief options 

What resources do you use to stay up to date on the current standard of care for providing pain relief during IUD placement?

Appendix 3

*Complete Qualitative Analysis* 

Patient population perception of pain:

There's three places where I feel like the pain is most significant. One is tenaculum placement. It doesn't hurt for everybody, but when it does that's really uncomfortable, or using ringed forceps. Either of those can cause the same pain. The other one is if there's any difficulty, like any stenosis to the internal Os or to the cervical canal… dilation causes a lot of pain. That doesn't happen for everybody, but when it happens, that is a major pain point. And then the third one is mostly with the sound reaching the fundus. Those are the three areas that I find people have the most discomfort. The actual IUD doesn't seem to hurt as much as the sound entering the fundus.

I think in general, more often than not, patients will say to me, " I know this is going to be painful," right? “Or you know you know how painful is it? 

You know I've had patients who [do not want] an IUD because they are [worried about pain]. They  definitely are like, "I'm just not interested in that... I don't want any pain.”, but there is certainly an element to that that is like my friend had it, and it was  really painful, and there's no way [I wanted to be in that kind of pain].

I think sounding and when you actually  place the  IUD at the fundus… are commonly the most painful aspects to it. And you know tenaculum placement is another one. Some people tolerate [tenaculum placement] really well…and other patients don’t. I definitely think there's a big variation with tenaculum placement.

Patients [who] come in about an IUD, they've usually heard about it being painful from a friend or they've had a past experience with it being painful. Sometimes people are like, "I think I want the IUD, but I think it's really painful," or, "I've heard it's really painful." And so we end up talking about the kind of pain control options and their concerns in that way. Yes, [I find patient’s opt for other LARCs besides IUDs] all the time. Especially people who've had it before, and they're like, "I can never do that again." I'd say more commonly. People who've not had it before but have heard stories are sometimes willing to try and see how it goes. Among people who have had IUDs, probably like 50% of the time pain from the first procedure will preclude patients from getting it a second time. It's definitely not insignificant.

I don't know that I have a solution, but so many people who I think are really interested in IUDs and would be great IUD candidates are not interested in an IUD because of the pain.

I do think a big thing with IUD placement is what the patient's experience of pain in a gynecological area has been in the past. So I do think a lot of patients do manage pretty okay just because, unfortunately, women have had to experience that pain in some way.

I tell patients that you know everyone's experience of pain is totally unique and subjective and don't compare yourself to your friends. There's many different levels of pain relief.

I tell patients, like I do these all the time, and it's a wide spectrum. And not everyone's on that really terrible, painful spectrum. Majority of people say that was not so bad. Yeah. I think a lot of people are getting those fears because of anecdotal experiences that they've heard from friends or family members who maybe did have a horrible, horrible experience.

I mean, if we could do anything to just change the stereotype so patients could come in a little more neutral about it and then just allow themselves to have their own experience as opposed to coming in with it with this big expectation of severe pain.

[My patients] are so anxious. They tell me. Some patients I have had…to really convince them that I'm not going to hurt them.

Use of misoprostol: 

I will be honest and say that is something that I do very rarely, but when I do, first of all, I don't see increased success in getting through the Os and I don't see any correlation between the miso and decreased pain for the patient. But my sample size is really small. I mean, at most I'm prescribing it like once a year. You know, maybe a couple times a year for that purpose.

[I do] not [use misoprostol] routinely. If people have had a failed IUD insertion, then we offer misoprostol for the second attempt…The only time I'm using Miso is if I'm doing a post-abortion IUD where they may have gotten miso before the procedural abortion. But for a standalone IUD procedure, the evidence for me has really fallen out of favor. So I never use it.

Not routinely, not as a first-line routine thing. But if for some reason there's challenges with passing anything through the OS, then yes. I can't remember the last time I actually needed to use it. I just have it as a backup option, but I can't remember. I really don't use it frequently.

I never use misoprostol. A miso-prepped cervix… is gushy and weird and very prone to trauma. Especially if you're using a traumatic tenaculum, you can rip right through it.

Provider training:

I did not receive training in providing analgesic when I was learning how to perform IUD insertions. Not at all. 

Nope, [I did] not [receive training] at all. Not during school or during my initial onboarding. It wasn't even discussed as an option. The only time it was really brought up was for, of course, our patients who are getting an in clinic abortion and then an IUD right after. But that's a different situation.

Because I did a complex family planning fellowship… the answer to that is yes [I did receive training], but I would say in residency and medical school, no, other than just [learning] a paracervical block, but the [paracervical block] wasn’t very prominent back then. 

Yes, I did [receive training]. Not formal training, but kind of on-the-go training in the clinic…during residency. And maybe a little bit in med school, but definitely more volume in residency. The way that I had always seen [pain relief being provided] was 800 milligrams of ibuprofen right before the procedure. And then a heating pad was…the go-to. And so that's something that I've carried forward.

I did not [receive training] during medical school. During residency, I'd say partial. In terms of our training in office procedure, like just an office-based IUD insertion, there was a little bit of discussion about pain relief, but it was much more focused on did they take the ibuprofen and not any of the other [pain relief options] that I talked about.

In residency, I was the one providing the training… after going out of my way to get training on my own. So what we had was an annual lecture and hands-on workshop using kiwis to practice injecting, which is super fun, and with a video and a hands-on component. So that's what we did. 

Pain relief education with abortion training

I learned about using a paracervical block for providing abortions and not the IUD insertion itself. Now I will say that I did learn that in the past, if you have a patient who has a very difficult IUD insertion you might refer them to somebody for something like a paracervical blocks or sedation. But it was never something that was offered to us as a skill that we could learn or that was appropriate to be offered to all patients.

I do a lot of post-abortion IUDs, and all of those people get a paracervical block for their abortion.

I do a ton of IUDs post-procedural abortion, and their sort of block is already on board for that, for the abortion portion of it.

So if someone's getting a post-abortion IUD, they will have had a paracervical block because they got one before their abortion.

I think that I'm thinking about the way that we do abortions, we always offer moderate sedation, which is a combination of fentanyl and Versed.

But the majority of my learning around IUD insertions was through abortion training…And so post-abortion IUD insertion. In that case, you're not just talking about pain relief for the IUD insertion. The IUD is like a little thing that's happening at the very end, whereas you're mostly doing the pain relief for the abortion procedure

I only learned paracervical block as part of abortion procedures. So that's how I learned to actually do it.

I learned [paracervical blocks] from an abortion provider. So that's kind of where my perspective on this is coming from and my comfort with it is mostly because I'm trained in abortion. So I'm very comfortable. And I'm comfortable doing it blind. I'll do a non-sounded IUD insertion blind. But that is an advanced skill that I do not recommend most people do unless you do it like every day. 

Paracervical block usage:

So I wouldn't say I use it routinely. I do definitely use it. With paracervical blocks, they can help for patients who have that pain with the dilation, like getting through stenotic Os, but it doesn't seem to really do much for that fundal discomfort that they have from the sound. In most patients, that is the  most uncomfortable part. And having a paracervical block placed is not fun. You know it's quite uncomfortable. So it's something that I mentioned as an option, right? But I typically only recommend it for patients who have difficulty getting through the cervix.

About 50% of my folks get a paracervical block.

If I  have a patient who I know is really nervous, I can sense that… I offer Valium for placement. I [also] offer paracervical block for placement and discuss with patients the options, risks, and benefits of them, of those two things in particular [paracervical blocks and Valium].

What I tell folks, the paracervical block itself hurts as well. And you know it can be sort of double the length of the procedure because the paracervical block takes just as long if not longer to do than to place the IUD itself. If you do have the paracervical block on board, then the IUD placement itself is better. It's not only anecdotal, there's also data to show the paracervical block does decrease the pain. And it does. And we see it. I do tons of IUDs on the paracervical block, and they do much better than people who don't have a paracervical block for the actual IUD placement.

 I see a huge variety in people's reactions to paracervical block. Some people are like, "Oh, okay. That sucks, but it's worth it for me because I am so scared about the IUD insertion." And I like knowing that I'm going to have a little bit of numbing on board. And other people are needlephobic and they're like, "Don't even talk to me about a needle in my cervix. Get away from me." You know what I mean?

It's very clear that paracervical block is helpful. It's just a matter of the risk-benefit of the pain associated with doing the block and anxiety associated with doing the block itself.

I think that paracervical block has its downsides too because it's not painless. And so there is this like, "Well, the block is just as painful as the IUD, so there's no reason to really offer it." And I think that is a bias there that while it's not untrue, it's also not the complete story.

I believe one of the major barriers is that it's not that the paracervical block doesn't work. Some providers' attitudes towards it are that their anecdotal experience is that the block itself is just as painful [as the IUD insertion].

Even when you offer a paracervical block to patients, there are many who opt out of it, and that's totally okay. And many opt out because for a lot of different reasons, But I think that I think many patients appreciate the offering.

If you can put an IUD in, you can use a paracervical block. If you're training people to put IUDs in, train them to be confident and comfortable providing a paracervical block. I don't think that should be like, "Oh, yeah, I'm trained in IUDs, but not a paracervical block." No. They should go hand in hand, actually and they don't in our American medical training system. 

The other option [I offer] is a paracervical block, which has been shown to reduce pain. I'd say that most of my patients opt to not have a paracervical block when I've offered it, but I routinely use them in abortion care. 

I do not typically [offer paracervical blocks] in my practice.

I've read some studies where paracervical blocks aren't always very helpful for patients, where it doesn't necessarily treat the pain, and that it simply prolongs the procedure. And some people's cervixes are pretty sensitive. And having your cervix…touched more than it should, can make the patient really uncomfortable.

We had noticed that the OB/GYNs pretty routinely give paracervical blocks for IUD insertions [and]at a minimum offer it. [We see OBGYNs] using it more frequently. And so that's why we're all kind of debating right now if we want to kind of do it like the family medicine way or the OB-GYN way.

I feel like even though you can do a paracervical block very quickly, just the additional seconds that that adds to the procedure when you could have just moved ahead and completed the procedure, I feel like it kind of slows things down.

The injection technique [with a paracervical] in the cervix is also really, really important. Providers who say the paracervical block doesn't work…. they're just not doing it right.I think the more polite way of saying that is that people have not been taught to do a paracervical block that is effective and efficient. They think it is in and of itself a painful intervention that is not effective, which makes sense why they wouldn't use something that they think hurts as bad as the procedure and doesn't work. The reason [providers] think that is that they're doing insufficient…blocks. They haven't been trained correctly. So the correct technique to do a paracervical block takes a little bit more time and it requires some comfort with continuous infiltration as a technique.

In my experience the paracervical block works… 99% of the time. In the 1% it doesn’t, I can fix it. Typical problems with paracervical blocks are [providers] not anesthetizing the tenaculum site, using a two-shot block instead of a four-shot block, using cold lidocaine, stuff like that. And it's all very easily fixable once you know the proper technique.

I very frequently have patients who are interested in [paracervical block] after I've described the procedure. I think there's something about needles that just really will freak patients out. And so I don't get a lot of buy-in….patients are often the most nervous for the needle. They'll ask like, "When's the needle coming? When's the needle coming?" Meaning the paracervical block. Similarly, I think that the anxiety around needles like needle phobia is so real that it's really hard for me.

Barriers to access

And sometimes that's just a scheduling thing, right? Because we do pull from so many different clinics. So often the clinic they're seeing me at is different from their home clinic. And then they'll call and reschedule. And you know it can be logistically different. Or people just don't want any side effects with the meso you know because meso can have nausea and vomiting and diarrhea and just you know can have some unpleasant side effects. So people are sort of like, "No, thanks." you know I don't want that.

The barrier [for light to moderate sedation] is more so the clinic's capacity. So like the clinic that I trained at in residency, you couldn't have done that because you have to have staff who are trained because it's technically like conscious sedation. So you have to have staff who are trained. So if that were the case, I would have to refer out.

Our medical center uses the Epic electronic medical record. And so there's something called MyChart that's attached to that. And our patients tend to be quite active on MyChart and they can message us and ask questions. And so it's really easy to just initiate a patient message. It's a bit of a clunky process right now. We just sort of groom our own schedule maybe the day before. And if we catch that there's an IUD procedure on there, then we'll go ahead and click and send it's a smart phrase that we have.

So it's like a templated thing just being like, "Here's all the prep about preparing and pain relief and everything." And so most of the time we catch them, sometimes they get missed. So sometimes we just didn't catch it. What we tried to do before when we had more staffing was actually to have a dedicated nurse who would groom the schedule like a week in advance and actually call and message the patients to prepare them for the appointment, including discussing pain relief. So that's what I would love to have, but we don't have staffing for that right now.

I think the biggest barriers, like what I said earlier, are just sort of like logistical and staff unfamiliarity with how to get things assembled.

Potential lose to follow up:

We don't have any anesthesiologists in our offices. But anytime we're discussing, with a patient who has a concern about pain, I offer to do it under sedation. But that would mean we wouldn't be able to do it on the same day. I think the biggest barrier we have for pain relief options for IUDs is that we're not really able to... We want to give patients what they want that same day. It's not ideal to make them come back, which makes it trickier to offer options like IV sedation and even intravaginal lidocaine gel, or anything like that. So you know that stuff is something that I struggle with a lot as a provider because I want my patients to have all the options, but we're not always in a place where all the options are readily available. So we will talk to patients if that's a big concern. We tell them we don't have to do this today and we can have you come back, but for a lot of patients, that's just not possible. 

Lack of training

I can probably do something about this now that I have the DO behind my name." So I sat down with my PD and I was like, "I'm going to start doing paracervical blocks. How do I get to the point where this happens?" And she was like, "This is probably one of the first big headaches I gave her in residency." She was like, You want what now?" she was like, "Okay, well, you need to get the didactics on it, and I need to set up faculty training." And by January, my intern year, faculty was comfortable precepting me.

And I did a bunch, and I got the whole residency, and now it's standard of care at my residency site.

I think the majority of these procedures are done in the primary care setting, right? So by someone's PCP, not necessarily by an OB/GYN. But I think that there's this thought that this is like a specialty procedure, but putting in an IUD is so straightforward. It doesn't need to be a specialty procedure.

Yes. I would say I received training from Bayer, who is the primary maker of Mirena, Skyla, those different forms of IUDs. And so they did talk about that as well as during my master's program, we talked about it, as well as my nurse practitioner clinical rotations. But primarily what's talked about is ibuprofen and Tylenol.

Adequate options for pain relief

I think a barrier is that there's not actually something that's really good out there to make it pain-free aside from deep sedation, which obviously then there are the barriers, just the risks of sedation is not risk-free from a medical complications perspective, the added cost, and then just the access.

Lack of patient education/stigma surrounding women’s pain

People have this acknowledgment that pain is a part of it, and that we just sort of have to if you want an IUD, you just sort of have to accept that. And I think that that can be an oversimplification of it. And therefore, probably less people are offered a paracervical block than could probably benefit from it.

doing a better job about educating patients to take the NSAIDs before their procedure because it doesn't work for the procedural pain, but it does work for post-procedural pain.

there is still so much stigma and anxiety related to procedures done on the uterus and cervix. People have a lot of fear and a lot of stigma and a lot of concern with that

I talked to one of my attendings about it, and I was like, "Why don't we do this? IUDs are arguably just as uncomfortable as a vasectomy." I think it just comes down to that we treat women's pain differently. Yeah She was like, "You know what? You're right." And so I have started to see a little bit of a culture shift around that in the last couple of years. I think more people are catching on to it.

I think a [major barrier] is probably that we view I think we view women's health and reproductive health procedures on female anatomy as somehow outside of the scope of routine outpatient care, like as a specialty thing. And I don't think it has to be. I think it can be in the primary care setting. And there are ways to integrate it into the primary care setting and make patients comfortable.

I would say most of [my patients] are terrified and have been traumatized in the past and have had their pain not taken seriously. I've been told like, "Oh, well, women can take it," or, "It's not that bad." My mentor who is extraordinary, who taught me a lot of this, always is fond of saying that I don't believe in unnecessary suffering… If we have the power to make it better, we should. 

Use of Nitrous oxide:

And I really want to highlight that nitrous is so underused in this field. We do so many painful procedures to women or people with a vulva and a vagina that we would never do to people with a penis without offering some form of pain relief.   I think of, you know, colposcopies, endometrial biopsies, IUDs and I really am so appreciative that you guys are looking at this issue because I think the more awareness around it the better. And I really want us to have nitrous available to us.

And I know this isn't the question you were asking, but this is why I feel so strongly that it would be such a benefit for everybody if we could have nitrous oxide in all of our clinics, because it's safe, there's no planning needed and no anesthesiologist needed.  And I feel like that's a big missed opportunity for our patients.

Right now, we're exploring a grant to bring nitrous oxide to a couple of our clinics.

Use of NSAIDs

So NSAIDs, we will recommend routinely. The problem is a lot of our patients come in and we don't know what they want ahead of time. So they're just like presenting to the office for an IUD. If we have a chance to have a conversation ahead of time,

[Patients’ are told at the time of scheduling by the call center or they can make an appointment online… scheduling asks them to take 800 milligrams of ibuprofen before their appointment so that hopefully they have that on board by the time that they get there.

That's our only standard thing that's 100% for everyone.

So you know we have pretty good access, but I would say still the overwhelming majority of IUDs in our system are done just with an NSAID with ibuprofen.

doing a better job about educating patients to take the NSAIDs before their procedure because it doesn't work for the procedural pain, but it does work for post-procedural pain.

I usually encourage patients to take the Advil before they come so that it has time to work. But if not, I'll kind of order it there and have the nurses give it to the patient beforehand.

Sometimes I see my patients before we have the IUD insertion scheduled. And then if so, I will recommend they take usually ibuprofen 600 to 800 milligrams before they come in for their appointment. If they prefer Tylenol, I usually recommend about 1,000 milligrams.

Vast majority of my patients, though, have the IUD placement scheduled prior to me ever meeting them. So when they do come in, I do ask if they've taken anything like ibuprofen or Tylenol. A lot of the times they have not. So then I offered to provide them 600 milligrams of ibuprofen that we have in the office or Tylenol if they so shoes.

I'd say most patients manage very well with ibuprofen. I mean, and we're talking about prescription-strength ibuprofen. Like I'm not saying 200 or 400 milligrams. I'm saying like 600 to 800 milligrams. And then if they're still feeling uncomfortable afterwards, adding on Tylenol if needed. Okay.

We have them take some type of oral pain medication, usually NSAID or Tylenol.

We're basically just telling them that we recommend taking some type of NSAID or Tylenol. I think it specifically says ibuprofen 600 milligrams with some food before the appointment.

Use of atraumatic tenaculum

Atraumatic tenaculums. People don't use them, but they're great…it's a great tool…. I am such a huge fan of an atraumatic tenaculum because [I feel] they help reduce bleeding.

Use of ultrasound

Okay. So that is painful. In nulliparous, specifically, traversing instrumenting the cervix is painful, especially if you have to dilate a little bit. Sounding is painful and can be painful even with a block, which is why I just do a procedure with ultrasound and don't sound. I don't use the sound. I use a prep dilator to make sure that the cervix will accept the inserter.

So I don't sound. It's not necessary if you have an ultrasound and you're good at using it. But typically, if you're sounding correctly, it should feel like pressure and not pain. And then I warn people so when my patients do feel pain is about an hour after the procedure when the block wears off and they're still having some residual cramps. I tell them if they take ibuprofen when they leave the clinic, it's pretty minimal.

Use of Ativan/other

Oh, and I will also give pre procedure... but again because of the barriers I don't usually have time to do this, but when it's appropriate I will offer like an Ativan or something to take ahead of time and that seems to help.

if I have a patient who I know is really nervous, I can sense that you know I offer Valum for placement

And then two other things that I always offer patients with kind of variable interest. One is a Valium, a 10 milligram Valium, which I will only do in the circumstance of a patient has come in for a pre-IUD appointment… They come in and they're like, "I just want to talk about my options. They decide on the IUD." And then they're like, "I'm going to come back for that." So in that case, I'm able to consent them that day right when they're totally sober. And then if they're interested in the Valium, we can make sure that they have a driver to take them home because they can't drive with Valium. And the Valium is less pain relief and more kind of like anxiety relief. So it sometimes just causes patients to feel a little bit more comfortable during the procedure and more comfortable anxiety-wise leading up to the procedure.

Yeah. I would say that most people, if they can get the Valium, like make the accommodations for Valium, they do want the Valiium. And I actually got this idea from a vasectomist who I worked with who routinely gives Valium. And we routinely give Valium for vasectomies in general. And after working with him for a little bit when I was training, I was like, "Why don't we do this for IUDs?" I was never trained.

So I've also talked to some other people who, in their pain relief bucket, they use Xanax to give patients before they come in for procedures. Is that something that you routinely do or you find that your patients don't really need that necessarily? I don't do it routinely. I actually prefer for the patient to be alert and to be able to converse with me and tell me what's going on.

Use of sedation

then if a patient is really like, "I'm really scared. I don't want this. I'm really nervous." Then you know I offer to reschedule them for moderate sedation with an IUD placement, which we do offer in our setting.

And I also do offer sedation for them if they so choose, if that is something that they are really concerned about.

Satisfaction with current pain relief options

No, I'm not really. I'm satisfied with the options that are there, but I'm not satisfied with the barriers that come between these options and providing IUDs in actual real, life clinical practice.

I mean, I'm not satisfied with the pain options for really anything related to the cervix or uterus.

Are you currently satisfied with the available pain relief options for IUD insertions? I am. I mean, not that many patients require the sedation option. But at my clinic, if patients do choose to go under sedation, they are offered either oral anti-anxiety medication like Ativan just to really help relax them and essentially make them a little bit more sleepy during the procedure.

I'd say sort of or moderately, not fully, but moderately. I'm not really sure what else we could use. We work very closely with one of the national experts in pain relief for uterine procedures, and she's given us some ideas too. But I mean, I think it'd be interesting to explore topical pain relief on the cervix where you wouldn't have to be injecting something, but maybe that could help with tenaculum placement.

Non pharmagologic pain relief options

I really think it's important not to forget about the non-pharmacologic pain relief options that we do for our patients, like walking them through the procedure, being respectful, and having a support person available, whether it's a staff member or whoever the patient wants with them. I feel like those are good patient care things that we can do that make a huge difference in our patient's perception of pain. If we explain everything and we walk them through step by step, it makes such a big difference. So I think that's really important to remember that by itself is often enough for people.

So the biggest thing is actually feeling prepared for your appointment and trusting the provider that you're working with. And then I try to put patients both using sort of a trauma-informed approach, but I tell patients they're fully in control. They get to let us know if something's not working for them or they should let us know what they're experiencing in terms of pain. And I'm really just there to assist them with the process and they're really sort of taking charge. We practice some deep breathing and some sort of abdominal floor release breathing together… And then I ask them to sort of engage with that as they're in the procedure itself. So I talk about sort of like all the talking, what they call verbal anesthesia and the trust with the provider and feeling calm in the room.

Use of hot packs

We also use hot packs. So we use a disposable hot pack and we have patients fold that on the low belly. I think it's both really helpful to distract them and to give them something to do, but I think it also is helpful in terms of pain relief.

I'll provide them a hot pack on their stomach and just check in with them, make sure they're doing all right. If at any point they want me to stop or pause anything, I will.

And then a heating pad was kind of like the go-to. And so that's something that I've carried forward. And then plus or minus Miso. But then in residency, when Miso kind of fell out of favor, no longer using Miso. Still doing Advil, heating pad if available.
